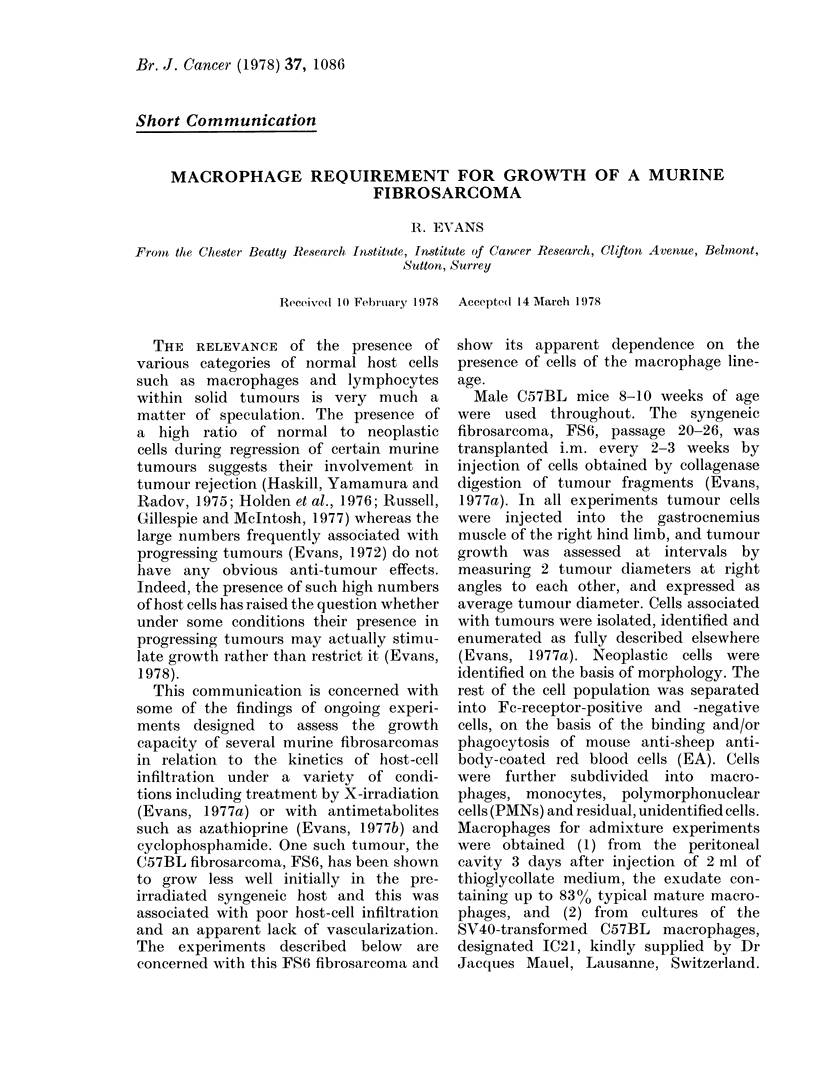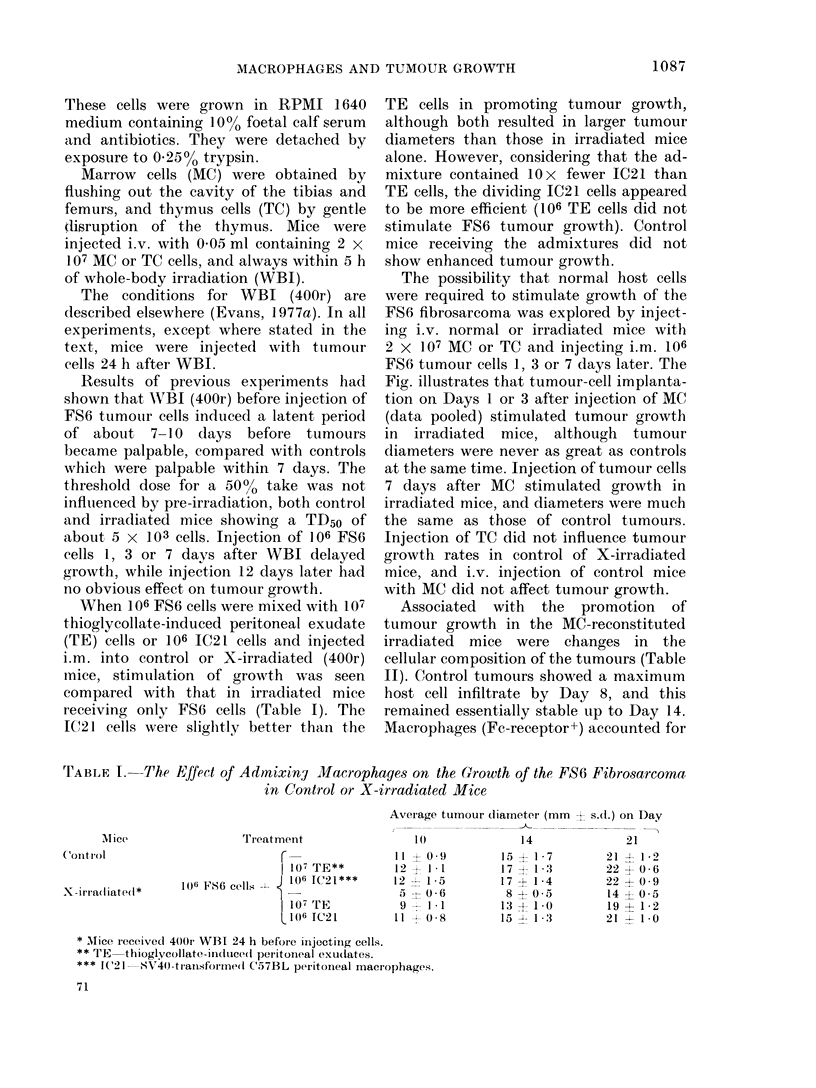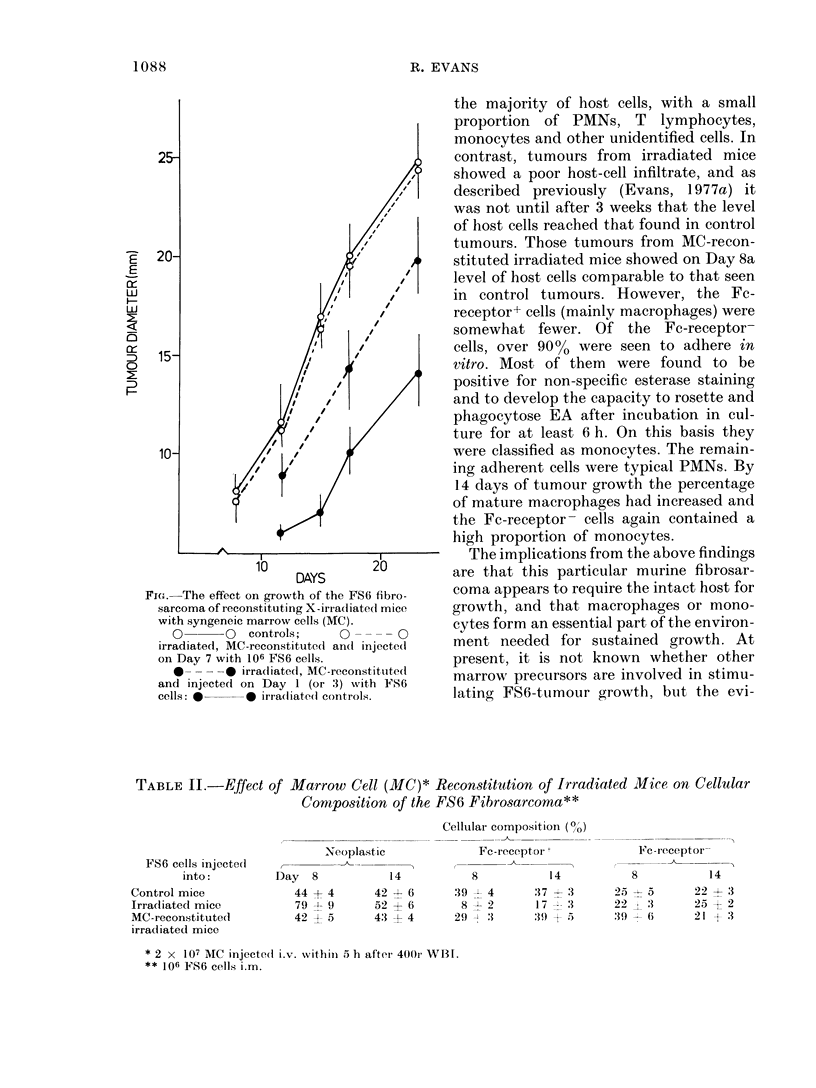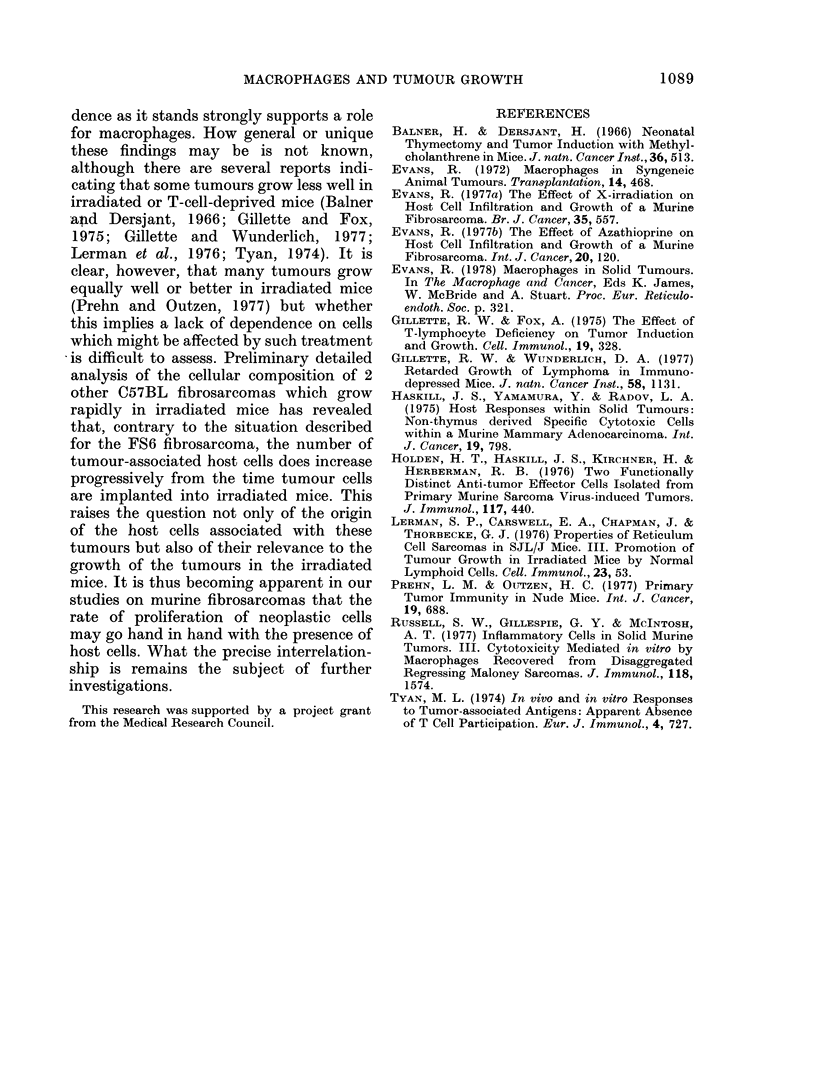# Macrophage requirement for growth of a murine fibrosarcoma.

**DOI:** 10.1038/bjc.1978.158

**Published:** 1978-06

**Authors:** R. Evans


Br. J. Cancer (1978) **37**, 1086

***Short Communication***

### MACROPHAGE REQUIREMENT FOR GROWTH OF A MURINE FIBROSARCOMA

R. EVANS

*From the Chester Beatty Research Institute, Institute of Cancer Research, Clifton Avenue, Belmont, Sutton, Surrey*

Received 10 February 1978 Accepted 14 March 1978

THE RELEVANCE of the presence of various categories of normal host cells such as macrophages and lymphocytes within solid tumours is very much a matter of speculation. The presence of a high ratio of normal to neoplastic cells during regression of certain murine tumours suggests their involvement in tumour rejection (Haskill, Yamamura and Radov, 1975; Holden *et al.*, 1976; Russell, Gillespie and McIntosh, 1977) whereas the large numbers frequently associated with progressing tumours (Evans, 1972) do not have any obvious anti-tumour effects. Indeed, the presence of such high numbers of host cells has raised the question whether under some conditions their presence in progressing tumours may actually stimulate growth rather than restrict it (Evans, 1978).

This communication is concerned with some of the findings of ongoing experiments designed to assess the growth capacity of several murine fibrosarcomas in relation to the kinetics of host-cell infiltration under a variety of conditions including treatment by X-irradiation (Evans, 1977*a*) or with antimetabolites such as azathioprine (Evans, 1977*b*) and cyclophosphamide. One such tumour, the C57BL fibrosarcoma, FS6, has been shown to grow less well initially in the pre-irradiated syngeneic host and this was associated with poor host-cell infiltration and an apparent lack of vascularization. The experiments described below are concerned with this FS6 fibrosarcoma and show its apparent dependence on the presence of cells of the macrophage lineage.

Male C57BL mice 8–10 weeks of age were used throughout. The syngeneic fibrosarcoma, FS6, passage 20–26, was transplanted i.m. every 2–3 weeks by injection of cells obtained by collagenase digestion of tumour fragments (Evans, 1977*a*). In all experiments tumour cells were injected into the gastrocnemius muscle of the right hind limb, and tumour growth was assessed at intervals by measuring 2 tumour diameters at right angles to each other, and expressed as average tumour diameter. Cells associated with tumours were isolated, identified and enumerated as fully described elsewhere (Evans, 1977*a*). Neoplastic cells were identified on the basis of morphology. The rest of the cell population was separated into Fc-receptor-positive and -negative cells, on the basis of the binding and/or phagocytosis of mouse anti-sheep antibody-coated red blood cells (EA). Cells were further subdivided into macrophages, monocytes, polymorphonuclear cells (PMNs) and residual, unidentified cells. Macrophages for admixture experiments were obtained (1) from the peritoneal cavity 3 days after injection of 2 ml of thioglycollate medium, the exudate containing up to 83% typical mature macrophages, and (2) from cultures of the SV40-transformed C57BL macrophages, designated IC21, kindly supplied by Dr Jacques Mauel, Lausanne, Switzerland.

These cells were grown in RPMI 1640 medium containing 10% foetal calf serum and antibiotics. They were detached by exposure to 0·25% trypsin.

Marrow cells (MC) were obtained by flushing out the cavity of the tibias and femurs, and thymus cells (TC) by gentle disruption of the thymus. Mice were injected i.v. with 0·05 ml containing $2 \times 10^7$ MC or TC cells, and always within 5 h of whole-body irradiation (WBI).

The conditions for WBI (400r) are described elsewhere (Evans, 1977*a*). In all experiments, except where stated in the text, mice were injected with tumour cells 24 h after WBI.

Results of previous experiments had shown that WBI (400r) before injection of FS6 tumour cells induced a latent period of about 7–10 days before tumours became palpable, compared with controls which were palpable within 7 days. The threshold dose for a 50% take was not influenced by pre-irradiation, both control and irradiated mice showing a $TD_{50}$ of about $5 \times 10^3$ cells. Injection of $10^6$ FS6 cells 1, 3 or 7 days after WBI delayed growth, while injection 12 days later had no obvious effect on tumour growth.

When $10^6$ FS6 cells were mixed with $10^7$ thioglycollate-induced peritoneal exudate (TE) cells or $10^6$ IC21 cells and injected i.m. into control or X-irradiated (400r) mice, stimulation of growth was seen compared with that in irradiated mice receiving only FS6 cells (Table I). The IC21 cells were slightly better than the TE cells in promoting tumour growth, although both resulted in larger tumour diameters than those in irradiated mice alone. However, considering that the admixture contained 10× fewer IC21 than TE cells, the dividing IC21 cells appeared to be more efficient ($10^6$ TE cells did not stimulate FS6 tumour growth). Control mice receiving the admixtures did not show enhanced tumour growth.

The possibility that normal host cells were required to stimulate growth of the FS6 fibrosarcoma was explored by injecting i.v. normal or irradiated mice with $2 \times 10^7$ MC or TC and injecting i.m. $10^6$ FS6 tumour cells 1, 3 or 7 days later. The Fig. illustrates that tumour-cell implantation on Days 1 or 3 after injection of MC (data pooled) stimulated tumour growth in irradiated mice, although tumour diameters were never as great as controls at the same time. Injection of tumour cells 7 days after MC stimulated growth in irradiated mice, and diameters were much the same as those of control tumours. Injection of TC did not influence tumour growth rates in control of X-irradiated mice, and i.v. injection of control mice with MC did not affect tumour growth.

Associated with the promotion of tumour growth in the MC-reconstituted irradiated mice were changes in the cellular composition of the tumours (Table II). Control tumours showed a maximum host cell infiltrate by Day 8, and this remained essentially stable up to Day 14. Macrophages (Fc-receptor+) accounted for

TABLE I.—*The Effect of Admixing Macrophages on the Growth of the FS6 Fibrosarcoma in Control or X-irradiated Mice*

| Mice | Treatment | | Average tumour diameter (mm ± s.d.) on Day 10 | 14 | 21 |
|---|---|---|---|---|---|
| Control | $10^6$ FS6 cells + | — | 11 ± 0·9 | 15 ± 1·7 | 21 ± 1·2 |
| | | $10^7$ TE** | 12 ± 1·1 | 17 ± 1·3 | 22 ± 0·6 |
| | | $10^6$ IC21*** | 12 ± 1·5 | 17 ± 1·4 | 22 ± 0·9 |
| X-irradiated* | | — | 5 ± 0·6 | 8 ± 0·5 | 14 ± 0·5 |
| | | $10^7$ TE | 9 ± 1·1 | 13 ± 1·0 | 19 ± 1·2 |
| | | $10^6$ IC21 | 11 ± 0·8 | 15 ± 1·3 | 21 ± 1·0 |

* Mice received 400r WBI 24 h before injecting cells.
** TE—thioglycollate-induced peritoneal exudates.
*** IC21—SV40-transformed C57BL peritoneal macrophages.

FIG.—The effect on growth of the FS6 fibrosarcoma of reconstituting X-irradiated mice with syngeneic marrow cells (MC).
○——○ controls; ○- - - -○ irradiated, MC-reconstituted and injected on Day 7 with $10^6$ FS6 cells.
●- - - -● irradiated, MC-reconstituted and injected on Day 1 (or 3) with FS6 cells: ●——● irradiated controls.

the majority of host cells, with a small proportion of PMNs, T lymphocytes, monocytes and other unidentified cells. In contrast, tumours from irradiated mice showed a poor host-cell infiltrate, and as described previously (Evans, 1977*a*) it was not until after 3 weeks that the level of host cells reached that found in control tumours. Those tumours from MC-reconstituted irradiated mice showed on Day 8a level of host cells comparable to that seen in control tumours. However, the Fc-receptor$^+$ cells (mainly macrophages) were somewhat fewer. Of the Fc-receptor$^-$ cells, over 90% were seen to adhere *in vitro*. Most of them were found to be positive for non-specific esterase staining and to develop the capacity to rosette and phagocytose EA after incubation in culture for at least 6 h. On this basis they were classified as monocytes. The remaining adherent cells were typical PMNs. By 14 days of tumour growth the percentage of mature macrophages had increased and the Fc-receptor$^-$ cells again contained a high proportion of monocytes.

The implications from the above findings are that this particular murine fibrosarcoma appears to require the intact host for growth, and that macrophages or monocytes form an essential part of the environment needed for sustained growth. At present, it is not known whether other marrow precursors are involved in stimulating FS6-tumour growth, but the evi-

TABLE II.—*Effect of Marrow Cell (MC)* Reconstitution of Irradiated Mice on Cellular Composition of the FS6 Fibrosarcoma***

| FS6 cells injected into: | Cellular composition (%) | | | | | |
|---|---|---|---|---|---|---|
| | Neoplastic | | Fc-receptor$^+$ | | Fc-receptor$^-$ | |
| | Day 8 | 14 | 8 | 14 | 8 | 14 |
| Control mice | 44 ± 4 | 42 ± 6 | 39 ± 4 | 37 ± 3 | 25 ± 5 | 22 ± 3 |
| Irradiated mice | 79 ± 9 | 52 ± 6 | 8 ± 2 | 17 ± 3 | 22 ± 3 | 25 ± 2 |
| MC-reconstituted irradiated mice | 42 ± 5 | 43 ± 4 | 29 ± 3 | 39 ± 5 | 39 ± 6 | 21 ± 3 |

* 2 × $10^7$ MC injected i.v. within 5 h after 400r WBI.
** $10^6$ FS6 cells i.m.

dence as it stands strongly supports a role for macrophages. How general or unique these findings may be is not known, although there are several reports indicating that some tumours grow less well in irradiated or T-cell-deprived mice (Balner and Dersjant, 1966; Gillette and Fox, 1975; Gillette and Wunderlich, 1977; Lerman *et al.*, 1976; Tyan, 1974). It is clear, however, that many tumours grow equally well or better in irradiated mice (Prehn and Outzen, 1977) but whether this implies a lack of dependence on cells which might be affected by such treatment is difficult to assess. Preliminary detailed analysis of the cellular composition of 2 other C57BL fibrosarcomas which grow rapidly in irradiated mice has revealed that, contrary to the situation described for the FS6 fibrosarcoma, the number of tumour-associated host cells does increase progressively from the time tumour cells are implanted into irradiated mice. This raises the question not only of the origin of the host cells associated with these tumours but also of their relevance to the growth of the tumours in the irradiated mice. It is thus becoming apparent in our studies on murine fibrosarcomas that the rate of proliferation of neoplastic cells may go hand in hand with the presence of host cells. What the precise interrelationship is remains the subject of further investigations.

This research was supported by a project grant from the Medical Research Council.

### REFERENCES

BALNER, H. & DERSJANT, H. (1966) Neonatal Thymectomy and Tumor Induction with Methylcholanthrene in Mice. *J. natn. Cancer Inst.*, **36**, 513.

EVANS, R. (1972) Macrophages in Syngeneic Animal Tumours. *Transplantation*, **14**, 468.

EVANS, R. (1977*a*) The Effect of X-irradiation on Host Cell Infiltration and Growth of a Murine Fibrosarcoma. *Br. J. Cancer*, **35**, 557.

EVANS, R. (1977*b*) The Effect of Azathioprine on Host Cell Infiltration and Growth of a Murine Fibrosarcoma. *Int. J. Cancer*, **20**, 120.

EVANS, R. (1978) Macrophages in Solid Tumours. In *The Macrophage and Cancer*, Eds K. James, W. McBride and A. Stuart. *Proc. Eur. Reticuloendoth. Soc.* p. 321.

GILLETTE, R. W. & FOX, A. (1975) The Effect of T-lymphocyte Deficiency on Tumor Induction and Growth. *Cell. Immunol.*, **19**, 328.

GILLETTE, R. W. & WUNDERLICH, D. A. (1977) Retarded Growth of Lymphoma in Immunodepressed Mice. *J. natn. Cancer Inst.*, **58**, 1131.

HASKILL, J. S., YAMAMURA, Y. & RADOV, L. A. (1975) Host Responses within Solid Tumours: Non-thymus derived Specific Cytotoxic Cells within a Murine Mammary Adenocarcinoma. *Int. J. Cancer*, **19**, 798.

HOLDEN, H. T., HASKILL, J. S., KIRCHNER, H. & HERBERMAN, R. B. (1976) Two Functionally Distinct Anti-tumor Effector Cells Isolated from Primary Murine Sarcoma Virus-induced Tumors. *J. Immunol.*, **117**, 440.

LERMAN, S. P., CARSWELL, E. A., CHAPMAN, J. & THORBECKE, G. J. (1976) Properties of Reticulum Cell Sarcomas in SJL/J Mice. III. Promotion of Tumour Growth in Irradiated Mice by Normal Lymphoid Cells. *Cell. Immunol.*, **23**, 53.

PREHN, L. M. & OUTZEN, H. C. (1977) Primary Tumor Immunity in Nude Mice. *Int. J. Cancer*, **19**, 688.

RUSSELL, S. W., GILLESPIE, G. Y. & MCINTOSH, A. T. (1977) Inflammatory Cells in Solid Murine Tumors. III. Cytotoxicity Mediated *in vitro* by Macrophages Recovered from Disaggregated Regressing Maloney Sarcomas. *J. Immunol.*, **118**, 1574.

TYAN, M. L. (1974) *In vivo* and *in vitro* Responses to Tumor-associated Antigens: Apparent Absence of T Cell Participation. *Eur. J. Immunol.*, **4**, 727.